# Ischemia induces different levels of hypoxia inducible factor-1α protein expression in interneurons and pyramidal neurons

**DOI:** 10.1186/2051-5960-2-51

**Published:** 2014-05-05

**Authors:** Prabhu Ramamoorthy, Honglian Shi

**Affiliations:** Department of Pharmacology and Toxicology, School of Pharmacy, University of Kansas, 5064 Malott, Lawrence, KS 66045 USA

## Abstract

**Introduction:**

Pyramidal (glutamatergic) neurons and interneurons are morphologically and functionally well defined in the central nervous system. Although it is known that glutamatergic neurons undergo immediate cell death whereas interneurons are insensitive or survive longer during cerebral ischemia, the protection mechanisms responsible for this interneuronal survival are not well understood. Hypoxia inducible factor-1 (HIF-1) plays an important role in protecting neurons from hypoxic/ischemic insults. Here, we studied the expression of HIF-1α, the regulatable subunit of HIF-1, in the different neuronal phenotypes under *in vitro* and *in vivo* ischemia.

**Results:**

In a primary cortical culture, HIF-1α expression was observed in neuronal somata after hypoxia (1% oxygen) in the presence of 5 or 25 mM glucose but not under normoxia (21% oxygen). Interestingly, only certain MAP2-positive neurons containing round somata (interneuron-like morphology) co-localized with HIF-1α staining. Other neurons such as pyramidal-like neurons showed no expression of HIF-1α under either normoxia or hypoxia. The HIF-1α positive neurons were GAD65/67 positive, confirming that they were interneuron-type cells. The HIF-1α expressing GAD65/67-positive neurons also possessed high levels of glutathione. We further demonstrated that ischemia induced significant HIF-1α expression in interneurons but not in pyramidal neurons in a rat model of middle cerebral artery occlusion.

**Conclusion:**

These results suggest that HIF-1α protein expression induced by ischemia is neuron-type specific and that this specificity may be related to the intracellular level of glutathione (GSH).

## Introduction

Neurons can be classified into three major groups, pyramidal neurons responsible for glutamate release, interneurons with round cell bodies responsible for γ–aminobutyric acid (GABA) release, and spiny interneurons with small cell bodies that can release both glutamate and GABA. Interestingly, ischemia-mediated vulnerability differs among neuronal subpopulations. Interneurons are resistant to ischemia in striatum, cortex, and hippocampus, whereas pyramidal neurons undergo immediate cell death under certain ischemic conditions [1–8]. However, the molecular mechanism for neuronal type-specific resistance to ischemia is not well understood.

Hypoxia inducible factor-1 (HIF-1) is a transcriptional factor that plays a critical role in cellular adaptation to low oxygen levels. It is a heterodimer consisting of two subunits, α and β. HIF-1β is constitutively expressed; the oxygen level has no effect on its expression. The protein level of HIF-1α is highly regulated by oxygen tension [9]. Thus, the activity of HIF-1 is primarily determined by the expression of the subunit HIF-1α and not that of HIF-1β. During hypoxia, HIF-1α is stabilized, translocates to the nucleus, binds to HIF-1β, and initiates transcription. HIF-1 plays an important role in neuroprotection against ischemia by upregulating various growth factors such as vascular endothelial growth factor and erythropoietin. It has been shown that HIF-1α knockdown increases brain injury in a mouse model of transient focal cerebral ischemia [10]. Inhibition of proline hydroxylase (PHD), an enzyme that initiates the degradation of HIF-1α, protects against glutamate-induced damage in the rat hippocampus [11]. Moreover, it has been reported that HIF-1α expression can vary in different cells. For example, its stability and degradation is regulated in a cell-type-specific manner in carcinoma cell lines [12]. The expression of HIF-1α differs in hepatoma and primary endothelial cells due to different degradation mechanisms [13]. Degradation occurs mainly in the cytosol in HEPG2 cells and in both cytosol and nucleus in mouse brain endothelial cells [13]. Previous data from our laboratory demonstrate that HIF-1α stability requires a reducing environment during ischemia and that increases in glutathione (GSH) levels stabilize HIF-1α in cortical neurons [14], indicating that protein levels of HIF-1α may vary among cells with different redox statuses.

We hypothesized that HIF-1α was expressed differently in pyramidal neurons and interneurons during hypoxic conditions. To test this hypothesis, we studied and compared the cell-type-specific expression of HIF-1α in pyramidal neurons and interneurons in a primary cortical neuronal culture exposed to hypoxia and an animal model of cerebral ischemia. We demonstrated that, under ischemic conditions, HIF-1α expression was remarkably stable in interneurons when compared to pyramidal neurons. HIF-1α stability in interneurons was consistent with an increase in intracellular GSH levels, suggesting that interneurons contain a highly reducing environment that maintains HIF-1 stability and expression during ischemia.

## Materials and methods

### Isolation of neurons

Primary neuronal cultures of cerebral cortices were obtained from Sprague–Dawley (SD) rat brains (postnatal day 0 [P0] to P3). Cultures were prepared according to Brewer et al. [15] with slight modifications. Whole cerebral cortices were dissected and then incubated for 50 min in 0.12% trypsin at 37°C. After the incubation, cells were washed completely with Hank’s balanced salt solution (HBSS) four times and dissociated with a fire-polished glass pipette in dissociation medium (HBSS, 0.1% BSA and 8 mM MgCl_2_), pelleted by centrifugation at 4000 *g* for 4 min at room temperature (RT), dissociated in starter medium (DMEM containing 10% FCS) and plated on coverslips. Coverslips were pretreated by incubation with poly-D-lysine (0.01%) for 1 hour (hr), then rinsed with sterile distilled water four times and dried before the cells were plated. Cultures were kept at 37°C in 5% CO_2_ for 1 hr, flooded with starter medium and incubated overnight at 37°C in 5% CO_2_. After 24 hrs, the medium was replaced with culture medium (Neurobasal plus 2 mM glutamine and B27 supplement). Every 4-6 days, half of the medium was replaced with fresh culture medium. After 12-18 days of *in vitro* culture (DIV), neuronal cells were used for the experiments.

### *In vitro* hypoxia or cobalt chloride (CoCl_2_) treatment

Neuronal medium was removed and replaced with fresh, serum-free experimental medium (DMEM) containing 0, 5, or 25 mM glucose. For normoxia, neurons were incubated under 21% oxygen at 37°C for 3 hrs. For hypoxia, cells were maintained with 1% O_2_/5% CO_2_ balanced with N_2_ at 37°C for 3 or 5 hrs. Incubation periods were selected based on our previous studies [14]. Expression of HIF-1α was observed in the cultured neurons after 3- and 5-hr hypoxic treatments, and further studies were performed only with 3-hr hypoxic treatments. For CoCl_2_ treatment, cortical neurons were incubated with 0.3 mM CoCl_2_ at 37°C for 3 hrs under normoxic conditions [16].

### Middle cerebral artery occlusion (MCAO)

**Figure 1:**
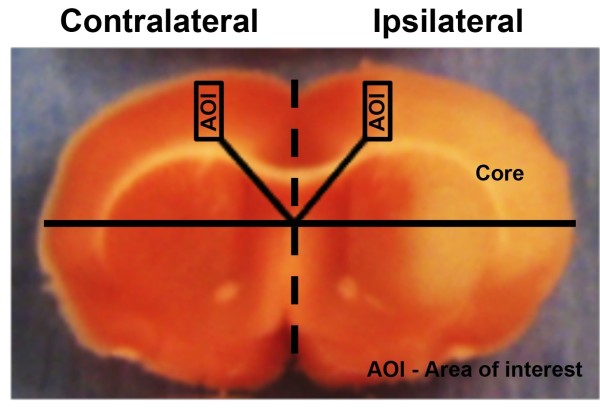
**A representative of TTC-stained rat brain coronal section.** The rectangular areas represent areas of interest (AOI) for imaging. The AOI in the ipsilateral cortex was selected at the peri-infarct tissue defined as the area between the ischemic core and normal tissue. The AOI of the corresponding area in the contralateral cortex was used as normal tissue (control).

Male SD rats (Charles River Laboratories, Wilmington, MA, USA) weighing between 250 g and 280 g were used in accordance with the Guide for the Care and Use of Laboratory Animals and with approval from the Institutional Animal Care and Use Committee. Middle cerebral ischemia/reperfusion was conducted according to the method of Longa et al. (1989), with some modification. Briefly, the right common carotid artery, including its bifurcation, was dissected, and the external carotid artery was divided, leaving a stump of 3–4 mm. The internal carotid artery was isolated, and the stump of the external carotid artery was reopened, and a 4.0 monofilament nylon suture with a slightly enlarged and rounded tip (Doccol Cooperation, Redlands, CA, USA) was inserted 17 mm through the internal carotid artery. Reperfusion was initiated by withdrawal of the filament at 90 min after occlusion. After surgery, the animals were allowed to recover from anesthesia while being given food and water *ad libitum*. Successful MCAO models were confirmed by 2, 3, 5-triphenyltetrazolium chloride staining (Figure 1) and by behavioral observation according to Rogers’ scales [17].

### *In vitro* immunocytochemistry

Immunocytochemistry was performed as described by Ramamoorthy et al. [18]. Briefly, cortical neurons were washed with phosphate buffered saline (PBS) and fixed with 4% paraformaldehyde in PBS for 20 min at RT. Fixed cells were washed with PBS and permeabilized using 0.3% Triton X-100 in PBS for 15 min at RT and incubated with blocking solution (PBS containing 0.05% Triton X-100 and 0.25% BSA) for 30 min at RT. Then, neurons were incubated with specific primary antibodies overnight at 4°C. Cells were washed with blocking solution for 4 × 15 min and incubated with an appropriate secondary antibody for 90 min at RT in the dark. Coverslips were washed with blocking solution and mounted with the temporary mounting medium Vectashield H 1000 (Vector Laboratories, Burlingame, CA). Fluorescent intensity was quantified with Image-Pro Plus 5.1 (Media Cybernetics). We randomly selected several cell free areas and calculated mean intensity as background intensity. We then determined fluorescent intensity in neuronal soma in normoxic and hypoxic conditions. The background intensity was subtracted from neuronal HIF-1α intensity. Changes in HIF-1α intensity caused by hypoxia were normalized to the normoxic level.

### *In vivo* immunocytochemistry

Rat brain was dissected after 90 min MCAO and 24 hr reperfusion. *In vivo* fixative was performed with 4% paraformaldehyde cardio perfusion. Then, brains were soaked in 4% paraformaldehyde overnight at RT and 30% sucrose for 48-72 hr at 4°C. Then, the brain was frozen using ice cold isopentane or liquid nitrogen for 2-3 min. Coronal brain sections were prepared from frozen brain sample using a cryostat. Slices were stained as described previously [18] with slight modifications. Briefly, after brain slices were washed with PBS and fixed (4% paraformaldehyde in PBS at RT) for 90 min, fixative was removed, and slices were washed with PBS and permeabilized in 1% Triton X-100 in PBS overnight at RT. Slices were incubated in blocking solution (PBS containing 2% BSA) overnight at 4°C and incubated for 48 hrs at 4°C with a primary antibody. Slices were washed in blocking solution for 15 min × 4 times and incubated with a secondary antibody for 4 hrs at RT. After washing, the slices were mounted in the temporary mounting medium Vectashield H1000. Control experiments were performed by omitting the primary antibody and incubating with secondary antibody alone.

Primary antibodies used were goat anti-HIF-1α (1-100; sc-8711; Santa Cruz Biotechnology), rabbit anti-HIF-1α (1-1000; 04-1006; Millipore Bioscience), mouse anti-HIF-1α (1-200; NB-100-123; Novus Biological), mouse anti-MAP2 (1:200; MAB378; Millipore Bioscience) and rabbit anti-GAD65/67 (1-400; AB1511; Millipore Bioscience). Secondary antibodies were donkey anti-goat Alexa 488 (1-100; Molecular Probes), goat anti-rabbit Alexa 488 (1-100; Molecular Probes), goat anti-mouse-FITC (1-50; Santa Cruz), donkey anti-mouse TRITC (1-50; Jackson ImmunoResearch) and donkey anti-rabbit DyLight 549 (1-2,500; Rockland). For double-staining experiments, antibodies were applied sequentially, starting with the anti-HIF-1α antibody. For antibody specificity, goat anti-HIF-1α antibody was incubated with HIF-1α blocking peptide (1-100; Santa Cruz) at RT for 2 hrs before staining the neurons for HIF-1α. Control experiments showed no significant bleed-through of the fluorescent labels or cross-reactivity between antibodies. As marked in Figure 1, the peri-infarct tissues in the ipsilateral cortex and the corresponding area in the contralateral cortex were selected as areas of interest. Images were obtained with a Leica DMI4000 microscope with a 40X objective and a Leica DFC340 FX Digital camera, using Leica LAS AF software. For quantification, the intensity of HIF-1α in the neuronal soma was randomly counted using MAP2- or GAD65/67-positive cell bodies. Fluorescent intensity was determined as described above.

### GSH staining

GSH was detected by using monochlorobimane (MCB) as reported by Chatterjee et al. [19]*.* Briefly, after hypoxic treatments, 0.1 mM of MCB was added directly to the neuronal petri dish and incubated for 20 min at 37°C. After the incubation, neurons were fixed and stained for specific neuronal markers using the method described above. The staining process was performed in the dark. MCB-GSH was visualized at an excitation wavelength of 380 nm and an emission wavelength of 480 nm using a fluorescence microscope. For quantification, MCB-GSH intensity was calculated for the neuronal soma. As it is impossible to measure MCB-GSH intensity in the same neurons before and after hypoxic treatments, different plates of neurons were treated with normoxia and hypoxia. Fluorescent intensity was determined as described above.

### Cell death

Dead or unhealthy neurons were counted visually by assessing morphological damage with the cytoskeletal protein MAP2 [20]. Healthy cells were identified by intact somata and processes, unhealthy cells were differentiated by swelling or bulging in the MAP2-positive processes and dead neurons were identified by disruption/breakdown in the MAP2 staining in both the cell body and processes.

### Statistical analysis

Image-Pro Plus, OriginPro7, and Excel were used for data analysis. One-way ANOVA and Student’s *t*-test were used for overall significance. Data are presented as the mean ± SD from at least 3 separate experiments. Differences were considered significant at p < 0.05.

## Results

### HIF-1α stability after hypoxia/ischemia is neuron-type-specific

**Figure 2:**
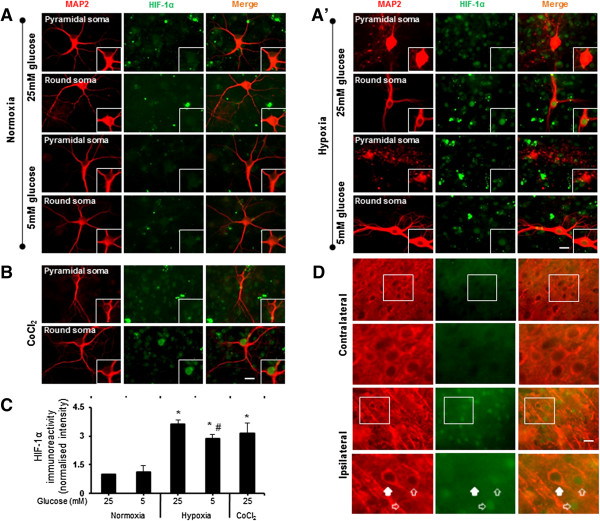
**HIF-1α expression in primary cortical neurons exposed to hypoxia/ischemia. A & A’)** Neurons were double-stained for HIF-1α and MAP2 in the presence of 5 and 25 mM glucose with and without hypoxia. HIF-1α expression in the somata was observed in cells with interneuron-like morphology after hypoxia. **B)** CoCl_2_ (0.3 mM) induced HIF-1α expression in cells with interneuron-like morphology. **C)** Quantification represents the increase in HIF-1α–ir staining (mean ± SD; 5-10 neurons quantified from each experiment, n = 3 experiments). **D)** *In vivo* brain slice shows a similar pattern of positive HIF-1α -ir in round soma (open arrow) and negative in neurons with pyramidal-like morphology (solid arrow) in the ipsilateral side. *p < 0.05, compared with normoxia (25 mM glucose), #p < 0.05, compared with hypoxia (25 mM glucose). Scale bars, 20 μm **(A, A’, B)**; 10 μm **(D)**.

Earlier studies have demonstrated that hypoxia/ischemia induces HIF-1α expression in cortical neurons. However, the cortical region of the brain contains multiple neuronal types with different survival rates during ischemia. We initially asked two questions. First, do all types of cortical neurons express HIF-1α in ischemia? Second, do only the surviving neurons express HIF-1α in ischemia? To address these questions, we examined the expression of HIF-1α in cultured cortical neurons exposed to hypoxia/ischemia. Under normoxic conditions, both round-soma-like and pyramidal-like neurons showed intact somata and processes; as expected, no expression of HIF-1α was seen (Figure 2A). Under hypoxic conditions, significant expression of HIF-1α was only observed in certain MAP2-positive neurons. Interestingly, all the neurons that expressed HIF-1α possessed round somata. This phenomenon was seen when cells were cultured in 5 or 25 mM glucose under 1% oxygen (Figure 2A’). The neurons with a pyramidal-like morphology showed no significant HIF-1α expression and had swollen somata and bulging/broken processes in both 5 and 25 mM glucose after hypoxia. Furthermore, when the neurons were treated with 0 mM glucose for 3 hrs, almost all the MAP2-positive neurons showed swelling or breakdown in their processes (unhealthy/dead) under normoxic conditions (data not shown). For further confirmation of the high expression of HIF-1α in the neurons with round somata, these experiments were repeated using cobalt chloride (CoCl_2_) treatment, a HIF-1α inducer. CoCl_2_-induced HIF-1α expression was found in the neurons that contained round somata (Figure 2B_,_ bottom panel) but not those with pyramidal-like morphology (Figure 2B, upper panel). Analysis of the total intensity of HIF-1α-immunoreactive (ir) neurons with round somata showed 3.0- and 3.5-fold increases in response to 5 mM and 25 mM glucose under hypoxia, respectively (Figure 2C).

To further confirm these findings, we examined HIF-1α expression in different neurons in the cortex of a rat MCAO model. Double staining of HIF-1α and MAP2 in coronal brain slices revealed that not all MAP2-ir neurons expressed HIF-1α in the cortical area. Similar to our observation in primary cultures, neurons with a round-soma-like morphology showed HIF-1α-ir in the ipsilateral region (open arrow), but no HIF-1α staining was observed in the contralateral region (Figure 2D). In addition, neurons with a pyramidal-like morphology showed no HIF-1α expression (Figure 2D solid arrow), confirming the *in vitro* data.

The above results demonstrated that the neuronal subpopulation containing round somata with intact cell bodies expressed HIF-1α under hypoxia. To assess neuronal survival under these conditions, we quantified the neurons subjected to *in vitro* ischemia using MAP2-ir staining. Neurons were divided into three categories: 1) intact cell body and processes (viable cells), 2) swollen cell body with swollen processes (unhealthy or dead), and 3) disrupted cell body and broken processes (dead). Under normoxia, 80-90% of neurons were viable in the presence of either 5 mM or 25 mM glucose, but in the presence of 0 mM glucose, the proportion of viable neurons dropped to 10%. When exposed to 1% oxygen, 70-80% of neurons were unhealthy or dead after 3 hrs in 5 or 25 mM glucose concentrations. There was no significant difference between normoxia and hypoxia when the cells were exposed to 0 mM glucose (Table 1). Further neuronal experiments were performed only with 25 mM glucose.

**Table 1:** **Hypoxia-induced neuronal viability in the presence of different glucose concentrations**

Treatments	Viability (%)
Normoxia	
25 mM glucose	90.18 ± 3.00
5 mM glucose	89.63 ± 2.72
0 mM glucose	10.43 ± 5.02*
Hypoxia	
25 mM glucose	30.84 ± 1.58*
5 mM glucose	22.27 ± 5.37*^#^
0 mM glucose	8.02 ± 5.96*^#^

Cell viability was estimated by integrity of the soma and processes. *p < 0.05, compared to 25 mM glucose, normoxia; ^#^p < 0.05, compared to 25 mM glucose, hypoxia (mean ± SD; n = 4 independent cultures).

### GAD65/67-positive neurons express HIF-1α following hypoxia/ischemia

**Figure 3:**
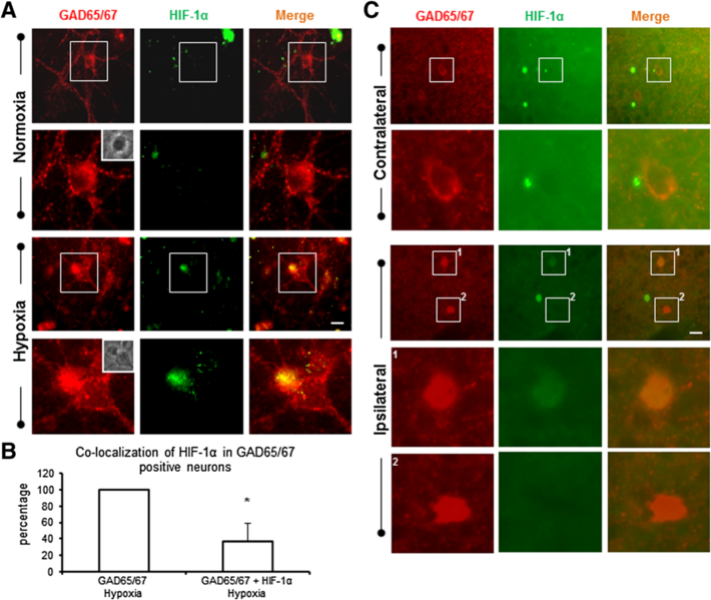
**GAD65/67-positive neurons expressed HIF-1α under hypoxic conditions. A)** HIF-1α expression co-localized with GAD65/67-ir in neurons exposed to hypoxia when compared to normoxia (upper panel) or GAD65/67-negative neurons in hypoxia (bottom panel, open arrow). **B)** Quantification shows the percentage of HIF-1α-expressing GAD65/67-positive neurons after hypoxia *in vitro* (mean ± SD; from n = 6 cultures). **C)** *In vivo* immunostaining illustrates HIF-1α-positive (bottom panel, solid arrow) and HIF-1α-negative (bottom panel, open arrow) in GAD65/67-ir neurons in the ipsilateral region, whereas the contralateral region shows no HIF-1α staining in GAD65/67-ir neurons (see Figure 1 for region selection). Scale bars, 10 μm **(A)**; 20 μm **(B)**.

To confirm that the round-soma neurons that expressed HIF-1α under hypoxia were GABAergic neurons, we double-stained neuronal cultures with antibodies against HIF-1α and GAD65/67 (interneuronal marker). As shown in Figure 3, under normoxia, neurons did not express HIF-1α, regardless of their GAD65/67 expression. Some neurons showed co-localization of HIF-1α and GAD65/67 under hypoxic conditions. No GAD65/67-negative cells were HIF-1α-ir whereas most GAD65/67-positive cells were HIF-1α-ir under hypoxia. We then quantified the percentage of HIF-1α-ir neurons showing coexpression of GAD65/67. These results demonstrated that 35-40% of GAD65/67-ir neurons were HIF-1α-ir (Figure 3B). For further confirmation, we co-stained brain slices from the rat model of MCAO with HIF-1α and GAD65/67 antibodies. These data demonstrated that HIF-1α-ir neurons co-localized with GAD65/67-ir neurons (Figure 3C, 1). It is noteworthy that not all GAD65/67-ir neurons in the brain slices expressed HIF-1α (Figure 3B, 2). These results confirmed that HIF-1α expression was specific to interneurons and that not all cortical interneurons expressed HIF-1α after ischemia.

### GSH stabilizes HIF-1α in interneurons after hypoxia/ischemia

**Figure 4:**
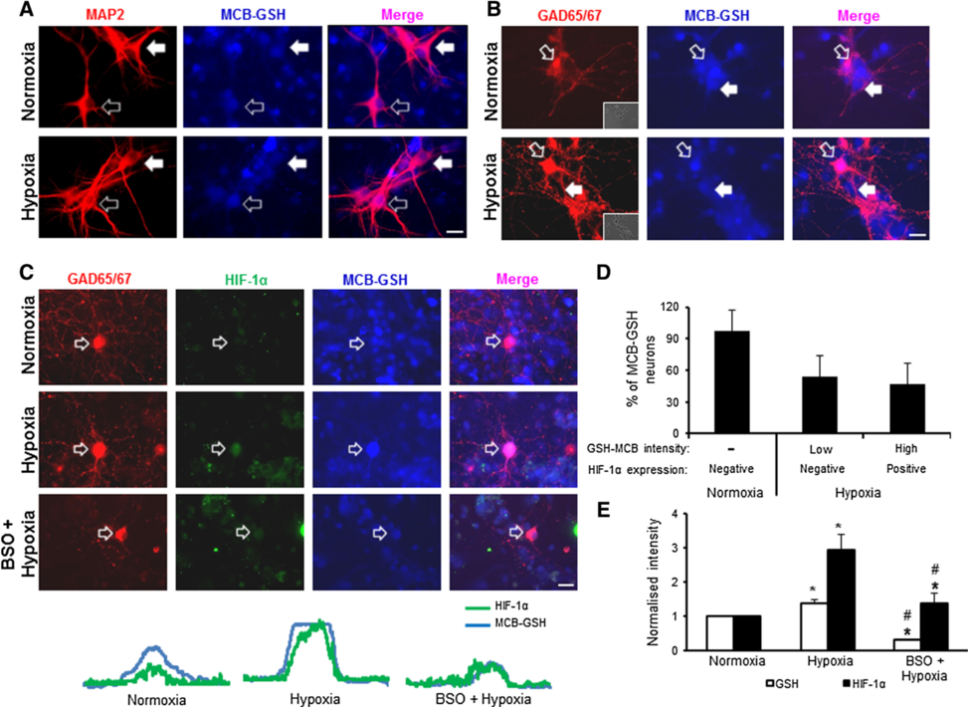
**Levels of GSH in cortical neurons exposed to hypoxia. A)** During hypoxia, GSH increased in MAP2-ir neurons with round somata (bottom panel, open arrow) and decreased in neurons with pyramidal-like morphology (upper panel, solid arrow). **B)** GSH increased in a subset of GAD65/67–ir neurons exposed to hypoxia (bottom panel, open arrow). **C)** Elevated HIF-1α expression in GAD65/67–ir neurons containing high levels of GSH when exposed to hypoxia (middle panel). BSO treatment decreased HIF-1α expression in GAD65/67-positive neurons during hypoxia. **D)** Percentages of HIF-1α-expressing GAD65/67-ir neurons expressing a high level of GSH and GAD65/67-ir neurons expressing a low levels of GSH and HIF-1α (mean ± SD; n = 3). The “low” level referred to the level of GSH in normoxic GAD65/67 neurons that was normalized to 1. The “high” level referred to the elevated GSH level in GAD65/67 neurons with a mean of 1.40 ± 0.10. **E)** Total MCB-GSH intensity in GAD65/67-positive neurons with and without BSO after hypoxia (mean ± SD; 8-10 neurons quantified from each experiment, n = 3 independent cultures). Scale bars, 20 μm.

We have previously reported that redox status plays an important role in HIF-1α expression in cortical neurons [14]. We postulated that GSH might play a role in regulating HIF-1α expression in different types of neurons. We therefore used MCB staining to evaluate levels of GSH in pyramidal neurons and interneurons exposed to hypoxia. Figure 4A shows that MCB-GSH intensity was equally expressed in all MAP2-ir neurons under normoxia. Interestingly, neurons with pyramidal-like morphology had less MCB-GSH staining under hypoxia than under normoxia. Neurons with interneuron-like morphology showed higher MCB-GSH staining in MAP2-ir neurons than the neurons with pyramidal-like somata after exposure to hypoxia. To confirm whether this increase in MCB-GSH was co-localized with GAD65/67 neurons, we stained the neurons for GAD65/67 after MCB treatments with and without hypoxia. Some of the GAD65/67-ir neurons showed an increase in the MCB-GSH signal under hypoxia compared to normoxia (Figure 4B, open arrow). GAD65/67-negative neurons with pyramidal-like somata showed reduced MCB-GSH staining during hypoxia (Figure 4B, lower panel, solid arrow). The inset in Figure 4B shows a phase-contrast image to confirm that the GAD65/67-negative neuron had a pyramidal-like soma. We then examined whether the neurons with an increase in MCB-GSH also expressed HIF-1α. This was done by co-staining the neurons with HIF-1α and GAD65/67 after MCB treatment. Normoxic neurons, including the GAD65/67-positive cells, did not show HIF-1α-ir, (Figure 4C, upper panel). Following hypoxia, neurons showed HIF-1α expression only in GAD65/67-ir neurons containing high levels of MCB-GSH (Figure 4C, middle panel and line profile). Interestingly, not all GAD65/67-ir neurons had increased levels of MCB-GSH under hypoxia. Quantification of the data revealed that almost 35-40% of GAD65/67-ir neurons were HIF-1α-ir and possessed high levels of MCB-GSH (Figure 4D). Furthermore, inhibition of GSH synthesis by buthionine sulfoximine (BSO) reduced the overall MCB-GSH intensity and suppressed HIF-1α expression in all GAD65/67-ir neurons exposed to hypoxia (Figure 4C, bottom panel). Quantitative data indicated that when the MCB-GSH level was reduced to 30-40% with BSO, the expression of HIF-1α in GAD65/67 positive neurons was completely inhibited (Figure 4E). These results suggest that differences in the level of GSH may be responsible for different levels of HIF-1α expression in hypoxic interneurons and pyramidal neurons.

## Discussion

The present study demonstrates for the first time that HIF-1α expression is cell-type-specific among cortical neurons in response to hypoxic/ischemic insults. Specifically, certain interneurons express a significantly higher level of HIF-1α protein than pyramidal neurons. Furthermore, the present results reveal that reduction in the GSH level might play a role in decrease in the HIF-1α level of the interneurons.

Previous reports have shown that the two major classes of neurotransmitter-containing neurons, pyramidal neurons and interneurons, are differentially affected during ischemia [3, 21]. Consistent with these reports, our results demonstrate that hypoxia induces swelling in the somata or disruption of processes in pyramidal neurons. In contrast, the interneurons show intact somata and processes during hypoxia. Pyramidal neurons and interneurons have different mechanisms for buffering intracellular calcium during hypoxia [22, 23]. Interneurons contain specific calcium-binding proteins (e.g., parvalbumin (PV) and calbindin (CaBP)) to regulate intracellular calcium for the survival of these cells [24, 8]. Because calcium is a critical damaging factor in ischemic neuronal death, these calcium-binding proteins might contribute to interneuronal survival. However, Freund et al. found that there was no consistent and systematic relationship between neuronal CaBP or PV content and ischemic vulnerability [25]. They and Crain et al. demonstrated that the majority of supragranular pyramidal cells that contained CaBP were the pyramidal cells most frequently degenerating after ischemia [26, 25]. In contrast, the density and distribution of non-pyramidal cells, mainly interneurons, containing CaBP or PV appeared qualitatively unchanged after ischemia [25, 26]. In addition, Larsson et al. have reported that increased neurotrophin signaling may provide neuroprotection in ischemic interneurons [27]. Our results clearly demonstrate that among the cortical neurons, GAD65/67-positive neurons, but not pyramidal neurons, express HIF-1α following hypoxia. These results indicate a novel pathway that may contribute to the resistance of interneurons to ischemia. Consistent with this concept, HIF-1 has been found to regulate transcription of the NTRK2 gene, which stimulates neurotrophin signaling [28].

It is worth noting that CoCl_2_ treatments also induce significant expression of HIF-1α in interneurons, whereas minimal expression is seen in neurons containing pyramidal-like somata and apical dendrites with intact cell bodies and processes. The dose of CoCl_2_ used in the present experiments does not cause cell death in either interneurons or pyramidal neurons. This indicates that the expression of HIF-1α in hypoxic interneurons is not necessarily a result of cell survival.

Many factors can regulate HIF-1α protein stability in an oxygen-independent manner. For example, heat shock protein 90 can stabilize HIF-1α, and receptor for activated kinase C (RACK1) can lead to HIF-1α ubiquitination and degradation [29]. Calcium might regulate HIF-1α expression in ischemic neurons. However, the available results are controversial or contradictory to each other. Calcineurin, a calcium-dependent protein phosphatase, may promote HIF-1α expression by de-phosphorylating RACK1 [30]. In contrast, it has been reported that lowering the intracellular calcium concentration activates HIF-1 through inhibition of hydroxylation of HIF-1α [31]. Unlike these results, Salnikow et al. reported that elevation of intracellular calcium neither induced the expression of HIF-1α protein nor stimulated HIF-1-dependent transcription [32]. However, Mottet et al. argued that elevated calcium levels after prolonged hypoxia increased extracellular signal-regulated kinase 1/2 (ERK 1/2) activation and increased HIF-1 transcriptional activity but did not induce HIF-1α accumulation [33]. Our results clearly show that GSH can stabilize HIF-1α protein in ischemic neurons. This is consistent with previous reports that HIF-1α can be strongly regulated by the redox environment [34–36], a mechanism that is of particular importance in the setting of ischemic brain injury because of the intrinsic changes in redox status. Although a more oxidizing environment has been suggested to stabilize HIF-1α in non-neuronal cells [37, 38], there is strong evidence supporting that a more reducing environment stabilizes HIF-1α, such as in COS7 cells [39], HeLa cells [40], HepG2 cells [41], MCF-7 cells [34], salmonid cells [42], renal medullary interstitial cells [43], and primary cultured neurons [14]. Reactive oxygen species can increase the activity of both 26S and 20S proteasomal degradation pathways under hypoxic conditions [44, 45]. Thus, it is highly possible that the presence of high levels of GSH decreases the activity of the 20S and 26S pathways and stabilizes HIF-1α protein in interneurons. Based on this discussion, GSH may not be the only factor contributing to HIF-1α expression in the two neuronal types. Other antioxidants that are able to reduce reactive oxygen species in the neurons may also contribute to the stabilization of HIF-1α protein.

The exact mechanism by which hypoxic interneurons maintain higher levels of GSH than hypoxic pyramidal neurons remains unclear. However, the following mechanisms may be involved. First, interneurons may generate lower amounts of free radicals than pyramidal neurons in hypoxia/ischemia. It is well established that ischemia causes excess free radical generation, mainly via mitochondrial dysfunction caused by excessive calcium. Interneurons can have lower levels of NMDA receptor activity by inhibiting Cys-299 of the NMDA receptor subunit NR2A [46]. This inactivation of glutamate receptors reduces the intracellular calcium and thus reduces free radical generation. Second, interneurons may have an enhanced defense system against oxidative stress. Interneurons have an enhanced and more efficient thioredoxin-2 system in detoxifying hydrogen peroxide, compared to pyramidal neurons [47]. Enhanced expression of antioxidants such as superoxide dismutase and Bcl-2 has been observed in ischemic interneurons [46]. In addition, the presence of nitric oxide may also help interneurons reduce free radical generation and protease activity [48, 46]. Overall, both low levels of free radical generation and enhanced levels of antioxidants may spare GSH in hypoxic interneurons.

## Conclusion

We report here that expression of HIF-1α is mainly detected in interneurons during hypoxia/ischemia. High levels of GSH in interneurons may play a role in maintaining the cellular environment for the expression of HIF-1α. These results provide essential information for understanding the pathophysiology of cerebral ischemia.

## Authors’ information

PR is an assistant research professor at University of Kansas Medical Center. HS is an associate professor in pharmacology, toxicology and neuroscience at School of Pharmacy, University of Kansas.

## Abbreviations

CaBP: Calbindin
GABA: γ–aminobutyric acid
GSH: Glutathione
HIF-1: Hypoxia inducible factor-1
HBSS: Hank’s balanced salt solution
MCAO: Middle cerebral artery occlusion
MCB: Monochlorobimane
PHD: Proline hydroxylase
RT: Room temperature.
